# Recurrent cryptococcal immune reconstitution inflammatory syndrome in an HIV-infected patient after anti-retroviral therapy: a case report

**DOI:** 10.1186/1476-0711-12-40

**Published:** 2013-12-20

**Authors:** Zhiliang Hu, Hongxia Wei, Fanqing Meng, Chuanjun Xu, Cong Cheng, Yongfeng Yang

**Affiliations:** 1 Department of Infectious Disease, The Second Affiliated Hospital of the Southeast University, Nanjing 210003, China; 2 Department of Pathology, Nanjing Drum Tower Hospital, The Affiliated Hospital of Nanjing University Medical School, Nanjing, China; 3 Department of radiology, The Second Affiliated Hospital of the Southeast University, Nanjing 210003, China

**Keywords:** Cryptococcal, Recurrent, Immune reconstitution inflammatory syndrome, Lymphadenitis

## Abstract

Cryptococcal immune reconstitution inflammatory syndrome (C-IRIS) in HIV-infected patients presents as a clinical worsening or new presentation of cryptococcal disease as a result of anti-retroviral therapy mediated immune restoration. Recurrent C-IRIS is a rare condition. Recently, recurrent C-IRIS involving the central nervous system, which is thought to require prolonged or alternative immunosuppressive therapy, has been described. Here, we present an unusual case of recurrent C-IRIS, sequentially involving the central nervous system and lymph nodes, in an HIV-infected patient after anti-retroviral therapy. While corticosteroids were used to control the inflammatory cerebral cryptococcomas, lymphadenitis that developed after cessation of corticosteroids resolved without additional immunosuppressive or anti-inflammatory drugs. This case suggests the possibility of site-specific recovery of pathogen-specific immune response after anti-retroviral therapy. In this condition, each episode of C-IRIS may be treated independently, and extended corticosteroids may not always be needed.

## Introduction

Cryptococcal immune reconstitution inflammatory syndrome (C-IRIS) presents as a clinical worsening or new presentation of cryptococcal disease after rapid immune restoration. In patients with human immunodeficiency virus (HIV)-1 infection, this immune restoration is driven by anti-retroviral therapy (ART). HIV-associated C-IRIS occurs in two forms: 1) paradoxical C-IRIS in patients diagnosed with cryptococcosis before ART who initially respond to antifungal therapy but then deteriorate or develop new clinical disease associated with ART-mediated immune restoration; 2) unmasking C-IRIS in patients who present with a first episode of cryptococcal disease with atypical inflammatory manifestations after ART [1,2].

The most common manifestation of HIV-associated C-IRIS is meningitis, accounting for around 70% of cases [1]. Lymphadenopathy as well as cerebral lesions have also been described [1]. Although C-IRIS occurs in 8 to 49% of HIV-infected patients with cryptococcal disease who initiate ART [1], recurrent C-IRIS is a rare condition. Recently, worsening of central nervous system (CNS) C-IRIS after cessation or tapering of corticosteroids has been described in 3 case reports [3-5]. The patients were successfully treated with additional courses of corticosteroids or other immunomodulatory drugs such as adalimumab or thalidomide [3-5]. This form of recurrent C-IRIS appears to be associated with an unusual inflammatory reaction that requires prolonged or alternative immunosuppressive therapy. Here, we present an unusual case of recurrent C-IRIS, sequentially involving CNS and lymph nodes, in an HIV-infected patient after ART. While corticosteroids were used to control the inflammatory cerebral cryptococcomas, the lymphadenitis that developed after cessation of corticosteroids resolved without additional immunosuppressive or anti-inflammatory drugs.

## Case report

**Figure 1:**
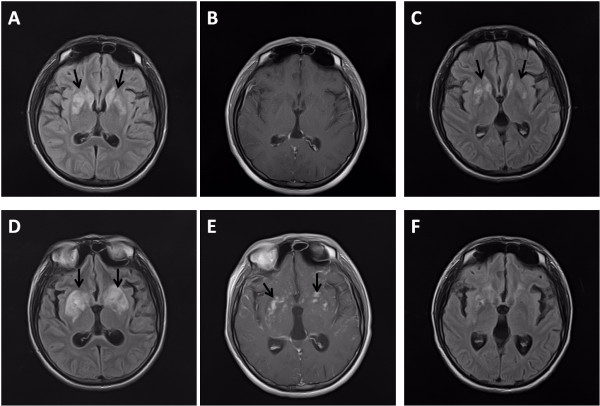
**Brain MRI of the patient.** Axial T2-FLAIR imaging obtained soon after admission showed abnormal hyperintensities in the bilateral basal ganglia (arrows in **A**) without enhancement **(B)**. After 7 weeks on anti-cryptococcal therapy, axial T2-FLAIR images showed partial resolution of the lesion (arrows in **C**). Twenty-five weeks after initiation of anti-retroviral therapy and while on fluconazole maintenance therapy, axial T2-FLAIR images showed abnormal hyperintensities in bilateral basal ganglia again (arrows in **D**) with irregular patchy enhancement (arrows in **E**). After 19 days on corticosteroids and intensified anti-cryptococcal therapy, T2-FLAIR images showed near complete resolution of brain lesions **(F)**.

A 19-year-old HIV-1-infected man was admitted to our hospital in late July 2011 because of fever and headache for two weeks and confusion for 4 days. His CD4 cell count was 0 cell/μL. Soon after his admission, he was confirmed to have cryptococcal meningitis. Cerebrospinal fluid (CSF) analysis demonstrated a white blood cell count of 32 cells/mm3, a glucose level of 1.4 mmol/L, a total protein level of 993.4 mg/L, and a positive India ink stain result. The baseline CSF fungal cell count under microscopy was 70 yeasts/μL. CSF and blood cultures were positive for Cryptococcus spp. Brain magnetic resonance imaging (MRI) revealed bilateral basal ganglia abnormalities consistent with cerebral cryptococcomas (Figure 1A, B). As per recommended clinical practice guidelines, he was treated with amphotericin B (AmB, 0.7 mg/kg per day intravenously) plus flucytosine (100 mg/kg per day in 4 divided doses) for 11 weeks, followed by fluconazole (400 mg per day) maintenance therapy [6]. CSF culture was sterile two weeks after initiation of anti-cryptococcal treatment. Follow-up lumbar puncture six weeks after initiation of anti-cryptococcal treatment revealed an improved CSF profile (white blood cell count of 2cells/mm3, a glucose level of 2.9 mmol/L and a total protein level of 147.8 mg/L). ART with lamivudine, stavudine and efavirenz was introduced in September 2011, after six weeks on anti-cryptococcal treatment. A follow-up brain MRI, obtained 7 weeks after initiation of anti-cryptococcal therapy revealed partial resolution of the cerebral cryptococcomas (Figure 1C). The patient was asymptomatic and discharged. Another brain MRI in February 2012 demonstrated near complete resolution of the cerebral cryptococcomas (see Additional file 1).

In March 2012, when the patient returned for a follow up visit in clinic, recurrent cerebral cryptococcomas (Figure 1D, E) were detected despite fluconazole prophylaxis (400 mg per day) and undetectable plasma HIV viral load with a CD4 cell count of 52 cells/μL. At that time, he was asymptomatic. CSF analysis demonstrated a white blood cell count of 11 cells/mm3, glucose level of 1.9 mmol/L, total protein level of 1260.2 mg/L, negative India ink stain and negative culture. CSF was further examined by a pathologist, and fungal cells as well as tumor cells were not identified. An interferon-gamma release assay (T-SPOT.TB) was also negative. Extensive laboratory studies revealed no active infection, including negative anti-toxoplasma antibody and undetectable cytomegalovirus viral load from the CSF and blood, although CSF cryptococcal antigen (CRAG) titer was 1:4 and serum CRAG titer 1:8. The recurrence of cerebral cryptococcomas was considered C-IRIS. The patient was observed without changing his treatment until he developed headache and dizziness in May 2013. At that point, anti-cryptococcal therapy was intensified with AmB, voriconazole (200 mg every 12 hours) and flucytosine for 6 weeks, followed by fluconazole (400 mg per day) maintenance therapy. ART was changed to lamivudine, tenofovir and raltegravir out of concern for drug interactions between voriconazole and efavirenz. Corticosteroids were co-administered and slowly tapered off over 6 months. His CD4 cell count increased to 108 cells/μL. Follow-up brain MRI confirmed near complete resolution of the brain lesions (Figure 1F).

**Figure 2:**
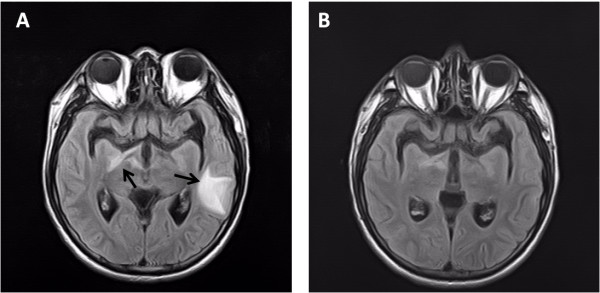
**Brain MRI of the patient after cessation of corticosteroid.** Axial T2-FLAIR images showed new cerebral lesions (arrows in **A**). After 16 days on intensified anti-cryptococcal therapy, T2-FLAIR demonstrated near complete resolution of cerebral lesions **(B)**.

**Figure 3:**
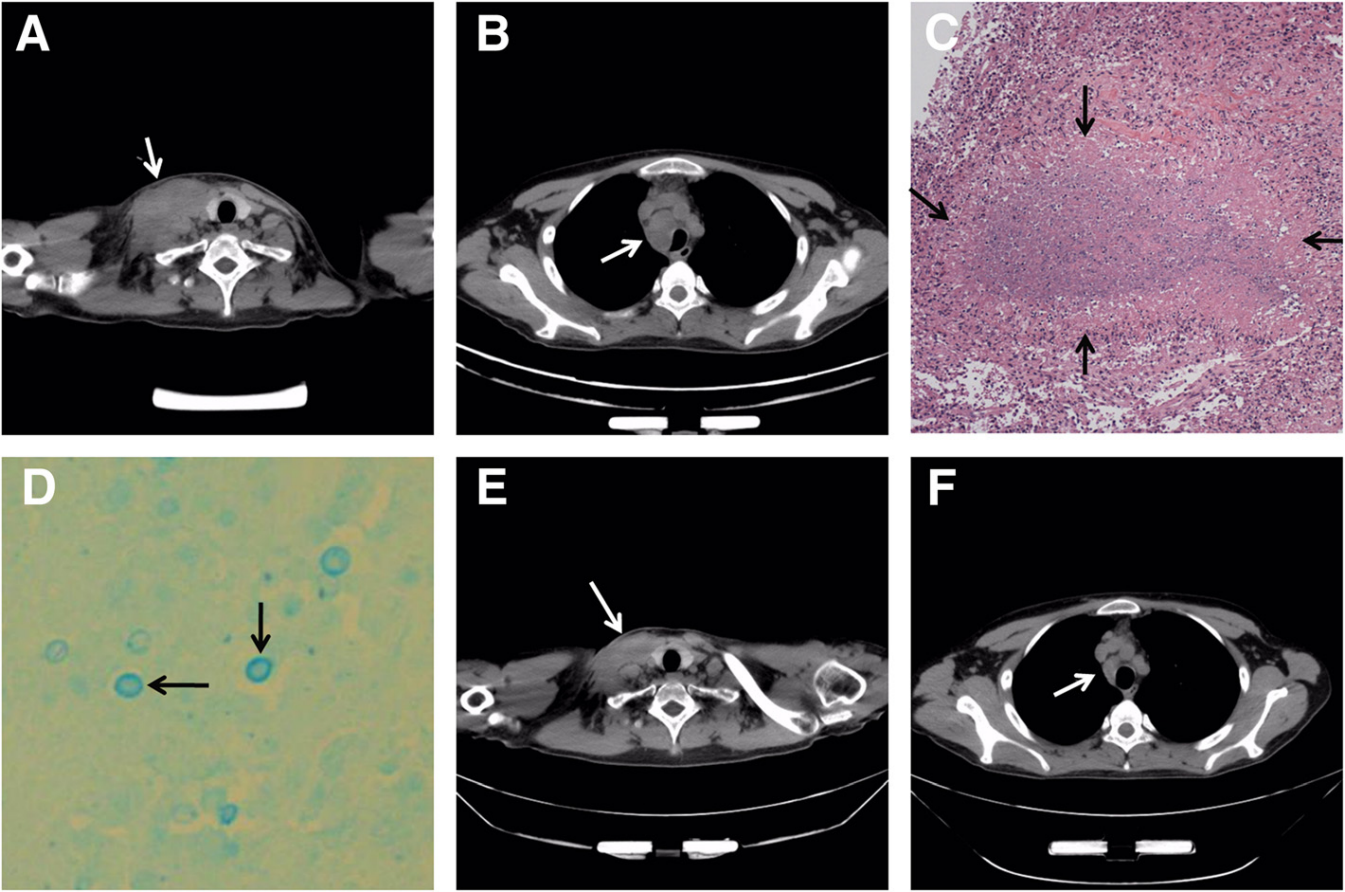
**Imaging and histological studies of the lymphadenopathy.** CT scan showing right cervical mass measuring 48 × 73 mm and trachea compression due to mediastinal lymphadenopathy (arrow in **A** and **B** respectively). Biopsy of the cervical mass revealed necrotizing granulomatous inflammation (arrows in **C**, haematoxylin and eosin stain, magnification × 40) and fungal structures (arrows in **D**, Alcian blue stain, magnification × 400). After 1 month of observation, repeat CT scan confirmed partial resolution of lymphadenopathy (arrows in **E** and **F**).

In December 2012, about 15 months after initiation of ART and while still on fluconazole maintenance therapy, a brain MRI demonstrated new brain lesions mainly involving the left temporal lobe (Figure 2A). CSF analysis demonstrated a white blood cell count of 4 cells/mm3, glucose level of 2.7 mmol/L, total protein level of 258 mg/L, negative India ink stain, negative cryptococcal antigen test and negative culture result. However, serum CRAG titer increased to ≥1:32. Despite undetectable plasma HIV viral load, his CD4 count decreased to 57 cells/μL. After re-intensification of anti-cryptococcal therapy with AmB, voriconazole and flucytosine for 19 days, to which the brain lesion responded favorably (Figure 2B), the patient developed a new, tender, protruding mass in the right neck in January 2013. The combination antifungal therapy was used for 6 weeks, followed by voriconazole (200 mg every 12 hours) for maintenance therapy. The mass did not improve with addition of amoxicillin/sulbactam and continued to progress. Subsequently, the patient complained of fever and cough. A computed tomography (CT) scan, obtained about eight weeks after re-intensification of anti-cryptococcal therapy, showed a mass in the right neck with compression of the trachea due to mediastinal lymphadenopathy (Figure 3A and B). A biopsy of the cervical mass revealed cryptococcal elements with necrotizing granulomatous inflammation (Figure 3C and D). Acid-fast bacilli were not identified in the biopsy specimen. Fungal culture of the biopsy specimen was negative. The patient refused corticosteroids because of adverse effects including weight gain and purple striae, and no new immunosuppressive or anti-inflammatory drugs were used. With anti-fungal therapy, the lymphadenopathy finally resolved (Figure 3E and F). In May 2013, the patient did not have any clinical manifestations associated with cryptococcal infection. The patient, who most recently had a CD4 cell count of 223 cells/μL in October 2013, has been closely followed and is asymptomatic.

## Discussions

Although there is no well-established diagnostic test for C-IRIS, useful consensus definitions published by the International Network for the Study of HIV-associated IRIS (INSHI) can be used to evaluate suspected cases [1]. These case definitions have been widely accept in primary studies for C-IRIS [7-9] and are readily used in our clinical practice. According to INSHI’s case definitions, encephalitis and/or meningitis with a negative CSF culture result and positive CSF cryptococcal antigen could be due to C-IRIS. Positive empiric response to corticosteroids further supports this diagnosis. Despite intensification of his anti-cryptococcal treatment, lymphadenitis occurred and continued to progress for a long time. Although cryptococcal elements were indentified in an inflamed lymph node, the biopsy culture was negative. Moreover, CSF analysis at the time the patient developed cryptococcal lymphadenitis was negative for cryptococcal elements, suggesting a very positive response to anti-cryptococcal therapy. Therefore, excessive inflammation observed in C-IRIS may be a contributing factor to lymphadenitis.

The pathogenesis of C-IRIS is still incompletely understood. Restoration of pathogen specific immune response after ART is thought to cause immunopathological lesions in tissues infected by that pathogen [10]. Nevertheless, CD4 cell counts are not included in the criteria for INSHI’s case definitions [1], as the number of CD4 cells measured in the periphery does not reflect the function and number of CD4 cells at the site of an opportunistic infection. Similarly, the recurrent C-IRIS sequentially involving CNS and lymph nodes implies the possibility of site-specific recovery of pathogen specific immune response. Unlike the management of the previously described recurrent C-IRIS that was more clearly regarded as a corticosteroid-dependent C-IRIS [3-5], the recurrent C-IRIS sequentially involving different sites may not always require extended corticosteroid therapy. Each episode of C-IRIS of this recurrent form may be treated independently.

## Conclusion

In conclusion, we present a case of recurrent C-IRIS, sequentially involving central nervous system and lymph nodes, in an HIV-infected patient after anti-retroviral therapy. This case suggests the possibility of site specific recovery of pathogen specific immune response after anti-retroviral therapy. In this condition, each episode of C-IRIS may be treated independently, and extended corticosteroid therapy may not always be needed.

## Consent

Written informed consent was obtained from the patient for publication of this case report and any accompanying images. A copy of the written consent is available for review by the Editor-in-Chief of this journal.

## Abbreviations

C-IRIS: Cryptococcal immune reconstitution inflammatory syndrome; HIV: Human immunodeficiency virus; ART: Anti-retroviral therapy; CNS: Central nervous system; CSF: Cerebrospinal fluid; MRI: Magnetic resonance imaging; AmB: Amphotericin B; CRAG: Cryptococcal antigen; CT: Computed tomography; INSHI: International Network for the study of HIV-associated IRIS.

## Competing interests

The authors declare that they have no competing interests.

## Authors’ contributions

HZ, WH, CC and YY have been involved in the clinical management of this patient. All imaging results were analyzed by X. MF performed the histopathology study in this case. This manuscript was initially drafted by HZ, and then revised by other authors in this study. All authors approved the final manuscript.

## Supplementary Material

Additional file 1 Brain MRI of the patient in February 2012 an brain MRI in February 2012 demonstrated near complete resolution of the cerebral cryptococcomas. Click here for file
