# Supplementary figures and images for: Recurrent cryptococcal immune reconstitution inflammatory syndrome in an HIV-infected patient after anti-retroviral therapy: a case report
Source: Ann Clin Microbiol Antimicrob. 2013 Dec 20;12:40. doi: 10.1186/1476-0711-12-40 (PMC3909341; doi:10.1186/1476-0711-12-40)

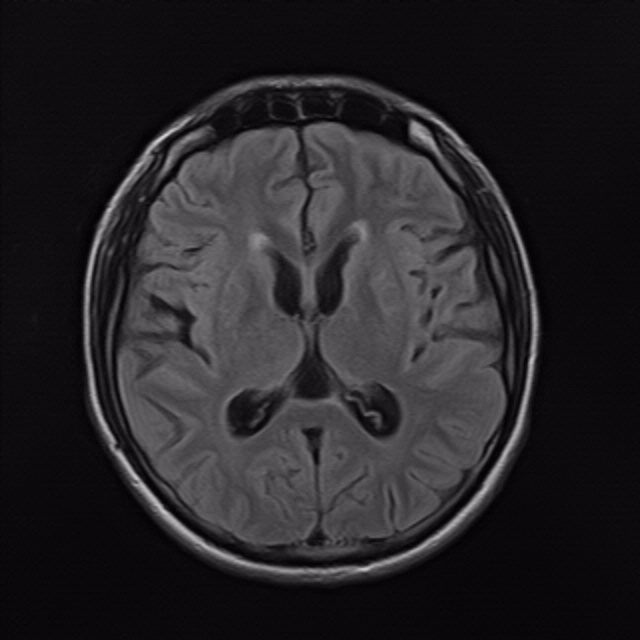

Supplement: Additional file 1 — Brain MRI of the patient in February 2012 an brain MRI in February 2012 demonstrated near complete resolution of the cerebral cryptococcomas. [file 1476-0711-12-40-S1.jpeg]
